# Glutamate and GABA in Microglia-Neuron Cross-Talk in Alzheimer’s Disease

**DOI:** 10.3390/ijms222111677

**Published:** 2021-10-28

**Authors:** Grzegorz A. Czapski, Joanna B. Strosznajder

**Affiliations:** Department of Cellular Signalling, Mossakowski Medical Research Institute, Polish Academy of Sciences, Pawińskiego 5, 02-106 Warsaw, Poland

**Keywords:** Alzheimer’s disease, glutamate, GABA, microglia, neurons, neurotransmission, neuroinflammation, signaling, neurodegeneration, therapeutic approaches

## Abstract

The physiological balance between excitation and inhibition in the brain is significantly affected in Alzheimer’s disease (AD). Several neuroactive compounds and their signaling pathways through various types of receptors are crucial in brain homeostasis, among them glutamate and γ-aminobutyric acid (GABA). Activation of microglial receptors regulates the immunological response of these cells, which in AD could be neuroprotective or neurotoxic. The novel research approaches revealed the complexity of microglial function, including the interplay with other cells during neuroinflammation and in the AD brain. The purpose of this review is to describe the role of several proteins and multiple receptors on microglia and neurons, and their involvement in a communication network between cells that could lead to different metabolic loops and cell death/survival. Our review is focused on the role of glutamatergic, GABAergic signaling in microglia–neuronal cross-talk in AD and neuroinflammation. Moreover, the significance of AD-related neurotoxic proteins in glutamate/GABA-mediated dialogue between microglia and neurons was analyzed in search of novel targets in neuroprotection, and advanced pharmacological approaches.

## 1. Introduction

Alzheimer’s disease (AD) is the major cause of cognitive impairment and dementia, which affects about 50 million people, and its prevalence was estimated to triple worldwide by 2050 [1]. The majority of AD cases are late-onset sporadic AD (SAD), whereas about 5–10% are early-onset familiar AD (FAD). Despite many years of intensive research and significant advances, we are still far from understanding of the pathogenesis SAD, but several genetic and environmental risk factors have been identified [2,3,4]. The amyloid cascade hypothesis, which dominated the field for about 30 years, suggested that the accumulation of amyloid β (Aβ) is a primary factor responsible for the pathological alterations [5]. Alternative hypotheses proposed other possible AD initiating factors, such as metabolic imbalance, infections, and toxins [2,6,7]. These factors, together with Aβ peptides and other proteins with altered conformation, affect several neurotransmitters and growth factors-dependent signaling pathways and communication networks between neurons, microglia, astrocytes, and other cells, leading to synaptic apoptosis and neuronal death.

However, all therapeutic strategies, including those based on anti-Aβ tactics, have failed to produce a reasonable effect, and to date there is no cure for AD. The inhibitors of acetylcholinesterase (AChE), and also an antagonist of the NMDA receptor (memantine), are commonly prescribed medications for AD, but these drugs do not stop the progression of the disease. Inhibitors of AChE, such as donepezil, galantamine, or rivastigmine, increase the level of acetylcholine, leading to normalization of cholinergic signaling, which is impaired in AD. Memantine blocks excessive glutamatergic signaling in the AD brain. Therefore, these drugs slow down the memory decline and maintain the ability to perform daily functions by stabilization of impaired neurotransmission in AD.

The imbalance between excitation and inhibition significantly contributes to AD pathology [8,9,10]. Alterations of the levels of glucose, glutamate, gamma-aminobutyric acid (GABA), and kynurenic acid may affect the homeostasis between major neurotransmitter systems in the brain, glutamate and GABA.

However, the function of glutamate and GABA is not limited to signal transmission between neurons, but they also play important roles in communications between neurons and microglia. Microglia, the only resident immune cells in the brain, have both immunological and non-immunological functions in the central nervous system (CNS). In a healthy brain, they play a prominent role in development, plasticity, cognition, and homeostasis. However, depletion, dysfunction, or undesirable activation of microglia may severely impair several signaling pathways, molecular processes, and communication with neurons, astrocytes, and as a consequence learning, memory, and other essential cognitive functions [11]. The growing body of evidence indicates their involvement in various aspects of the pathomechanism of AD. The novel research tools and experimental approaches reveal the heterogeneity of the microglial population, the complexity of these cells’ function, and dysfunction, and highlight the crucial role of neurotransmitters in tuning up microglial function [12,13,14,15,16,17]. Microglia express several receptors for neurotransmitters and neuroactive compounds, among them receptors for glutamate, GABA, acetylcholine, dopamine, adrenalin, ATP, and adenosine [18,19]. The aim of this article is to review the current knowledge on the relationship between microglia and neurons in the pathomechanism of AD. We will focus on the role of glutamatergic and GABAergic neurotransmitter systems in cross-talk between these cells in the brain, and on its impairment in the progression of AD. Moreover, based on this knowledge, the novel pharmacological therapeutic approaches will be presented.

## 2. The Role of Microglia in the Brain

Microglia arise from erythromyeloid precursors originating from the yolk sac in early embryogenesis [20,21]. The microglia migrate via the circulatory system and penetrate the developing CNS according to a specific spatiotemporal pattern, and in humans, this process was observed during the first two trimesters of gestation [22]. The analysis of rodent development demonstrated that in the postnatal period the number of microglial cells in the brain initially increases, and then their population begins declining, due to developmental apoptosis and decreased proliferation [23]. 

**Figure 1:**
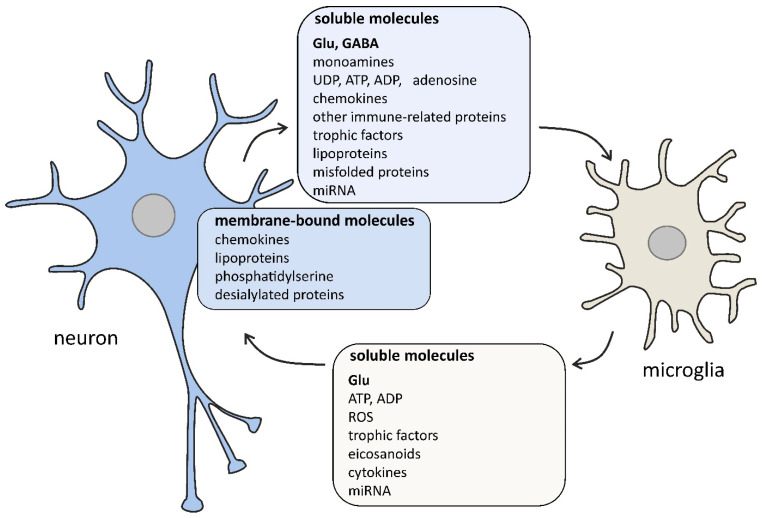
The selected signaling molecules, both cell surface-bound and soluble, that are involved in the cross-talk between neurons and microglia. The bidirectional communication between neurons and microglia is mediated by numerous molecules. Neurons affect microglial function by classical (glutamate, GABA, dopamine, serotonin, ATP) and non-classical neurotransmitters (UDP, ADP, adenosine), chemokines (membrane-bound and soluble CX3CL1), membrane-bound proteins (CD200, CD22, CD34, CD47), complement system components (C1q, C3), trophic factors (colony-stimulating factor-1, transforming growth factor β), lipoproteins (apolipoprotein E), membrane-bound phosphatidylserine, desialylated membrane proteins, misfolded proteins (amyloid β, α-synuclein), and miRNA (e.g., miR-124 and miR-9). Microglia affect neurons by releasing gliotransmitters (glutamate, ATP, ADP), reactive oxygen species (ROS; e.g., nitric oxide, superoxide), eicosanoids (prostaglandin E_2_), cytokines (interleukin 1β, tumor necrosis factor α), trophic factors (brain-derived neurotrophic factor), and miRNA (e.g., miR-146a). Some signaling pathways occur only under pathological conditions.

In a healthy brain, microglia play a key role in shaping and fine-tuning brain circuits; they control homeostasis and development via scavenging cellular debris, secretion of trophic factors, and monitoring synaptic development and activity. During perinatal development, microglia regulate neurogenesis and control synaptic density (synaptic pruning). In the adult brain, by secreting insulin-like growth factor 1 for cortical layer V, microglia provide neurotrophic support to neurons. Microglia represent 5–12% of all glial cells in rodent brains and 0.5–16% in humans, depending on the brain region [24,25,26]. It is important to underline that microglia are engaged also in learning and memory processes through the production and liberation of brain-derived growth or neurotrophic factors as nerve growth factor (NGF), brain-derived neurotrophic factor (BDNF), glial cell-derived neurotrophic factor (GDNF), insulin-like growth factor 1 (IGF-1), fibroblast growth factor (FGF), and colony-stimulating factor 1 (CSF1) (Figure 1) [27,28]. Additionally, microglia accumulate in brain regions with a high density of neuronal precursors and significantly contribute to the maintenance of embryonic progenitors [21].

Genomic, epigenomic, and transcriptomic analyses revealed that microglia are very different from other glial cells and tissue macrophages [29,30]. The signaling factors derived from the tissue microenvironment promote the specialized phenotype in the residing microglia population. The recent transcriptome-wide studies on human brain tissues based on analysis of transcriptomes of single cells shed light on the heterogeneity of the microglial population. Olah and co-workers identified some separate human microglial subpopulations (clusters) with specific gene expression patterns [12]. This microglial cluster architecture was present in all (17) tested persons. Importantly, one of these subpopulations (clusters) was significantly affected in AD, which suggests that a small fraction of brain microglia may be directly involved in the pathomechanism of AD [12]. Additionally, these data suggest also the need for understanding the specific function of each separate cluster of microglia and its contribution to the pathomechanism of AD. Moreover, because of phenotypic diversity, targeting microglia in AD is challenging and it is necessary to find an approach that does not suppress microglia overall, but instead modulates specific subpopulations or pathways. Adaptation of microglia/macrophages to local tissue microenvironment, including neuron-derived signal molecules, was shown to be mediated by epigenetic changes in chromatin [31]. These epigenetic mechanisms comprise histone methylation and acetylation, as well as miRNA. Several approaches targeting epigenetic regulation of microglia/macrophage function were proposed, including modulation of epigenetic code readers—bromodomain and extraterminal domain (BET) proteins [32,33,34,35]. 

## 3. Microglial Phenotype Polarization during Neuroinflammation/Neurodegeneration and the Role in Intercellular Communication

In a “resting” state, microglia very vigorously infiltrate the local microenvironment by extending and retracting motile processes equipped with a vast number of surface receptors. Detection of any specific or unspecific stimuli related to infection or tissue damage stimulates microglia to execute a respective response. The standard classification of the activation phenotype of microglia was borrowed from the nomenclature of macrophages, which, after stimulation in vitro, achieved M1 or M2 phenotype, depending on the stimulus. According to this classification, M1 (classic, proinflammatory, neurotoxic) microglia produce high amounts of proinflammatory mediators (cytokines, chemokines, and eicosanoids, including prostaglandins) and reactive oxygen/nitrogen species. Therefore, when overactivated, M1 microglia may be dangerous for own host’s tissues. On the contrary, M2 (alternative, anti-inflammatory, neuroprotective) microglia release anti-inflammatory cytokines and produce enzymes, such as arginase, which are necessary for tissue repair and regeneration. Later, additional variants were added to this list, such as M2a, M2b, M2c, and also M3, which has a role in the anti-inflammatory defense and tissue repair and can be stimulated by macrophage colony-stimulating factor (MCSF), interleukin-4 (IL-4) and IL-34 [36,37]. Recent studies analyzing transcriptomic and proteomic profiles clearly demonstrated that even this classification is a great oversimplification and is not relevant in categorizing microglia in vivo. In response to factors present in the tissue, activated microglia do not form steady subsets, but rather represent a broader functional repertoire of complex, even mixed, interpenetrating states, with M1-like and M2-like being just some near-border options [38,39]. 

The analysis of AD patients suggests that distinct neuroinflammatory microglial populations exist within the brain and that the neuroinflammatory status changes as the disease develops [12,40]. Microglia activation in neuroinflammation and AD is mediated by several complex signaling pathways [41,42]. Pathogen-associated molecular patterns (PAMPs) and danger-associated molecular patterns (DAMPs) have been demonstrated to mediate microglia activation and immune response via different pattern-recognition receptors (PRRs), including Toll-like receptors (TLRs) and nucleotide-binding and oligomerization domain (NOD)-like receptors (NLRs). Microglia, through complex mechanisms, are involved in the non-autonomous clearance of protein aggregates in the brain. This function of microglia is significantly changed in brain aging and AD and may lead to proteinopathy. Accumulation of Aβ and several other proteins with altered conformations is responsible for disturbances in communication between microglia and neurons and also with other cells in the brain. These processes lead to neuroinflammation/neurodegeneration, cell death, mood disorders, and severe dementia [42,43].

## 4. The Role of Aβ Peptides and Other Proteins with Altered Conformations in the Neuron–Microglia Dialogue in AD and Neuroinflammation

The neuropathological hallmarks of AD are the presence of extracellular senile plaques, which are mainly built of aggregates of Aβ and the neuronal presence of neurofibrillary tangles (NFT), consisting mostly of the hyperphosphorylated microtubule-associated protein (MAP) Tau. However, in AD brains, other proteinopathies exist with high frequency [44] and may also influence neurotransmitters signaling, including glutamate and GABA, and their role in the microglia–neuron communication/interaction [45,46,47,48]. Aβ peptides significantly influence glutamatergic and GABAergic neurotransmission and the level of these compounds and may be responsible for disturbances of learning and memory processes [49,50]. The proteins most frequently involved in various forms of dementia are amyloid-β (Aβ), MAP Tau, α-synuclein (α-Syn), and TAR DNA-binding protein 43 (TDP-43). These proteins may act intracellularly and also extracellularly, and their interaction could potentiate toxicity and may disturb the microglia–neuron dialogue and evoke additional effects, as a release of several cytokines, trophic factors, free radicals, and also altered proteins. These processes form some type of metabolic vicious circle—for example, neuron-derived extracellular Tau can induce microglia-mediated neuronal death [51]. Interaction of extracellular Aβ with α-Syn leads to irreversible molecular alterations in cells and to neuronal cells death [52]. There are several suggestions and indications on the role of altered proteins α-Syn and Tau in the transmission of protein pathology from cells to cells similarly to prions [53,54].

Moreover, these peptides and other altered proteins affect the concentration and signaling pathways of several neurotransmitters, such as serotonin, dopamine, purines, and acetylcholine, which at an early stage may influence mood and induce apathy, agitation, or depression. These symptoms are, in many cases, the first alterations in neurodegenerative disorders, including Alzheimer’s or Parkinson’s disease [55,56]. However, the significance of altered proteins in microglia–neuron communication during AD is not fully elucidated. Moreover, the relations between proteins with altered conformation and the glutamate and GABA in microglia–neuron cross-talk in AD should be better understood. 

Microglia play a crucial role in removing an excessive amount of Aβ and other altered proteins in AD. They may contribute to both the metabolism of Aβ and neuroinflammation. The presence of “activated” microglia in AD was originally reported by Alois Alzheimer in his report on Auguste D. in 1907 [57]. It was later hypothesized that the microglia are contributors to the pathomechanism of this disease via an autotoxic loop in which activation of microglia is primarily triggered by Aβ plaque deposition. This initial activation leads to further tissue damage, followed again by microglial activation, and thus, the process would continue in a vicious circle. The role of an altered amount of glutamate and GABA in such an autotoxic loop seems to be important.

The interest in microglia-powered neuroinflammation has increased in recent years, mainly because of genome-wide association studies (GWAS) done on large cohorts, counting thousands of patients, identifying new loci associated with the risk of developing a sporadic form of AD (*ATXN1*, *CD33*, *CLU*, *CR1*, *PICALM*, *BIN1*, *CD2AP*, *MS4A6A*/*MS4A4E*, *EPHA1*, *ABCA7*, *SHIP1*, *TREM2*, and an unidentified locus on chromosome 14 (GWA_14q31.2)) [58,59,60,61,62,63]. The effect of virtually all of these genetic factors on the pathomechanism of AD is associated with the regulation of the microglial function. For example, the triggering receptor expressed on myeloid cells 2 (TREM2) is involved in the phagocytosis of not only apoptotic neurons but also living neurons and synapses in neurodegeneration [64]. It was suggested that inhibition of microglial phagocytosis is sufficient to prevent inflammation-related neuronal death [65]. Recently, galectin-3 was found to be a specific novel endogenous TREM2 ligand [66]. Based on a study in the AD animal model, it was also suggested that TREM2 is responsible for the switch of microglia cells from the basal homeostatic and the intermediate state to disease-associated state and itself is an AD risk gene [67]. 

**Figure 2:**
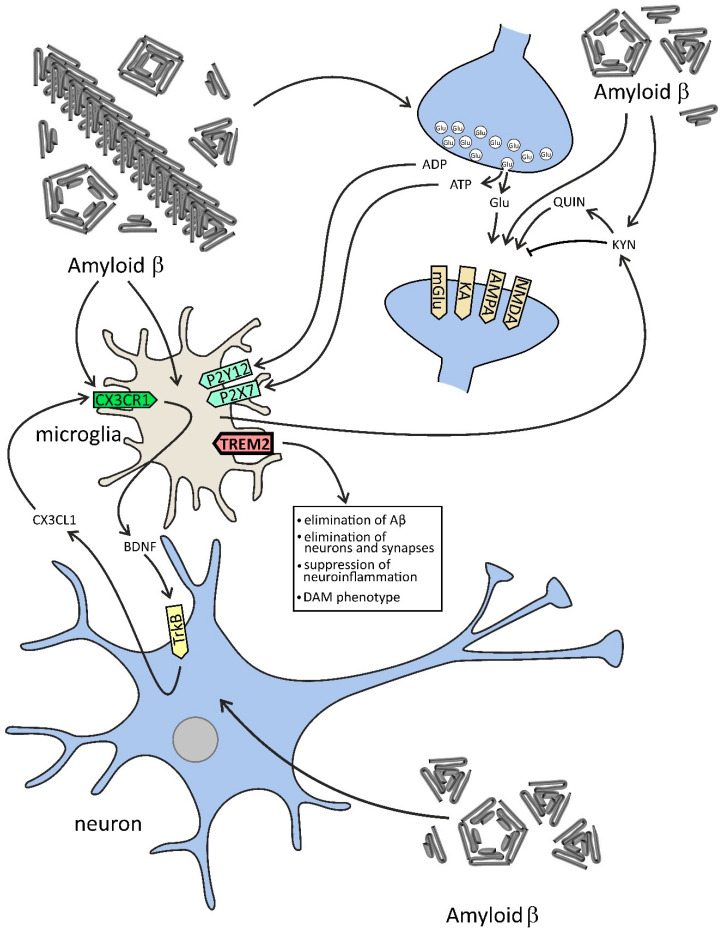
The effect of Aβ on selected components of microglia–neuron dialogue in AD. In this figure, the effect of Aβ on neurons and microglia is demonstrated. Aβ peptides evoke the release of glutamate and ATP from neurons, which activates neuronal NMDA and other receptors and in microglial cells exerts a significant effect on purinergic receptors. Aβ-activated microglia change tryptophan metabolism toward the kynurenine (KYN) pathway, which leads to an increase in the level of KYN and other molecules affecting glutamatergic signaling, such as quinolinic acid (QUIN), which may evoke excitotoxicity. Microglia concomitantly release neurotrophic factors, among them brain-derived neurotrophic factor (BDNF), leading to stimulation of tropomyosin receptor kinase B (TrkB) and release of chemokine (C-X3-C motif) ligand 1 (CX3CL1). The proper function of microglia is dependent on the TREM2 (triggering receptor expressed on myeloid cells 2) receptor, which is engaged in the clearance of Aβ and other pathological proteins, but also in the elimination of neurons and synapses, and in the suppression of neuroinflammation. TREM2 is also responsible for the switch of microglia phenotype to disease-associated state (disease-associated microglia, DAM).

The TREM2 receptor is also engaged in the clearance of Aβ and other pathological proteins; therefore, the alteration in its function impacts the microglia-driven response to Aβ and could be important in microglia–neuron interaction (Figure 2). In AD brain and in transgenic models of AD, microglia accumulate around Aβ plaques, but this does not occur in TREM2-deficient or TREM2-haploinsufficient mice [68,69,70]. However, TREM2 deficiency decreases the expression of several genes necessary for microglial activation, including inflammatory cytokines, trophic factors, and proteins related to phagocytosis. Importantly, the significant impact of systemic inflammation on cognitive decline and on glutamate and GABA level and transmission in AD was also shown [71,72,73,74,75]. Moreover, the data suggest that systemic factors, such as tumor necrosis factor α (TNF-α), may significantly contribute to neuroinflammatory signaling in the brain [76]. Some data support the hypothesis of a possible role of infection/inflammation in the etiopathogenesis of AD [77,78,79]. The association was demonstrated between the frequency of several bacterial and viral infections and the risk of AD [80]. Recently, *Porphyromonas gingivalis*, the keystone pathogen in chronic periodontitis, was suggested to be directly involved in the etiopathogenesis of sporadic AD through the release of neurotoxic gingipains [81,82]. 

## 5. Glutamatergic Signaling in Microglia–Neuron Communication in AD

The recent hypothesis of homeostasis network collapse suggests that the imbalance between excitation and inhibition in the CNS, leading to dysregulation of neuronal networks, might be an exacerbating or even causative factor in the etiopathology of AD [83,84,85,86]. In parallel with changes in the balance between inhibition and excitation of neurons, dysregulation of microglial function may occur. It is obvious that microglial cells, to efficiently perform their homeostatic function, are equipped with a set of receptors. Several receptors are necessary for continuously surveying the CNS for changes in homeostasis, including proteostasis, for detecting the presence of invading pathogens or the harmful processes inside the CNS. A comprehensive list of receptors expressed in microglia has been published recently [18,19,78].

Glial cells express many receptors for neurotransmitters and actively respond to neuronal activity. Recent data indicate that neurons and glia share some components of signaling pathways and the cross-talk between them plays a prominent role in the development and function of the CNS (Figure 1) [87,88].

Glutamate, the most important neurotransmitter in CNS, is a nonessential amino acid that is synthesized in neurons and glial cells from glucose and α-ketoglutarate and is easily distributed throughout the brain. Its level in CNS is not affected by peripheral organs because it does not cross the blood–brain barrier (BBA). The average concentration of glutamate in the brain is 10–12 mM, and in a synaptic vesicle, it exceeds 100 mM. However, the concentration of glutamate in the synaptic cleft is in the range of 0.6 µM up to 10 µM during presynaptic neuronal depolarization; therefore, in the brain’s extracellular compartment, it must be maintained at a low micromolar level. The gradient of glutamate in different cerebral compartments is coordinated by specific transporters and enzymes that are responsible for its metabolism.

Glutamate is the precursor of GABA, the other crucial neurotransmitter with opposite functions. The balance between these two neurotransmitter-mediated signaling pathways is fundamental for appropriate brain function. Glutamate, under physiological conditions, is released by a mechanism dependent on various types of stimuli from synaptic vesicles and transduces the information via specific ionotropic and metabotropic receptors. Several diverse types of signaling pathways lead the molecular information to the nucleus, and through the regulation of gene transcription, glutamate may influence the metabolism and function of the brain. One of the most important roles of glutamatergic transmission is regulation of cognition, learning, and memory through activation of long-term potentiation (LTP) or long-term depression (LTD) by calcium-dependent liberation of nitric oxide and arachidonic acid, and its different metabolites, as eicosanoids and docosanoids [89,90]. Recently, the molecular control of glutamatergic NMDA receptor-dependent GABAergic plasticity was described [91].

**Figure 3:**
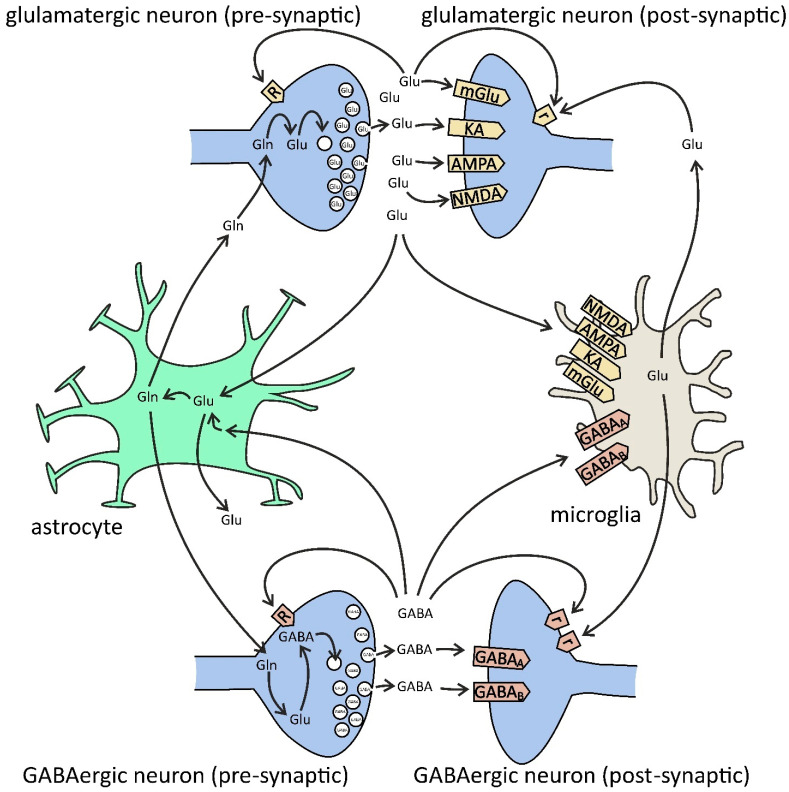
Glutamate (Glu) and GABA in glia–neuron cross-talk. Presynaptic [R] and extrasynaptic [r] receptors. Glutamate is synthesized from glutamine (Gln) by phosphate-activated glutaminase (PAG) and then is packed into synaptic vesicles by vesicular glutamate transporters (VGLUT1/2). After release into the synaptic cleft, glutamate binds to neuronal postsynaptic receptors for glutamate (ionotropic N-methyl-D-aspartate (NMDA), alpha-amino-3-hydroxy-5-methyl-4-isoxazolepropionate (AMPA), kainate (KA), and metabotropic mGluR1/5), to presynaptic receptors (ionotropic NMDA, AMPA, KA, and metabotropic mGluR7), and extrasynaptic receptors (mGluR2/3/4/8), but also to microglial receptors (NMDA, AMPA, KA, and mGluR2–8). Glutamate is also released by microglia via gap-junction hemichannels (e.g., Cx32) and by astrocytes via NMDA and purinergic P2X7 receptors, plasma membrane glutamate transporters, anion transporters, gap-junction hemichannels, and exocytosis. The major uptake of glutamate is performed by astrocytes via excitatory amino acid transporters (EAAT1/2). In astrocytes, glutamate is converted to glutamine by the glutamine synthetase (GS) pathway. Then, glutamine is transferred from astrocytes back to the neurons. GABA is synthesized from glutamate by glutamate decarboxylase (GAD) and then is packed into synaptic vesicles by the vesicular GABA transporter (VGAT). After release into the synaptic cleft, GABA binds to neuronal ionotropic GABA_A_ (pre-, post-, and extrasynaptic), GABA_c_ (pre- and postsynaptic), and metabotropic GABA_B_ (pre-, post-, and extrasynaptic) receptors, but also to microglial GABA_A/B_. Both presynaptic nerve terminals and surrounding glial cells are responsible for the uptake of GABA from extracellular space. In glia, GABA is converted to glutamine, and then it is transferred back to the neurons and converted to glutamate, and finally to GABA.

However, neuroinflammation and AD lead to aberrant neurotransmitters’ release not only from neuronal synapses but also by microglia and astrocytes (Figure 3 and Table 1). The excess amount of glutamate is rapidly removed by glial cells and by excitatory amino acid transporters (EAAT1, EAAT2). In astrocytes, glutamate is transformed to glutamine, which is released and taken up by neuronal presynaptic part and metabolized to glutamate, which is subsequently accumulated in synaptic vesicles by vesicular glutamate transporters (VGLUT1/VGLUT2), and then released in the process of neurotransmission in very well operated tri- or tetrapartite synapse. The most important glutamate transporter is GLT-1/EAAT2, which is responsible for about 90% of forebrain uptake of glutamate and its homeostasis in the adult brain [92].

Glutamate released from synaptic vesicles exerts its role through activation of several ionotropic and metabotropic glutamate receptors localized mainly in the postsynaptic part of synapsis. However, its excessive release, also from glial cells, may affect several extrasynaptic receptors in neurons and microglia, and it may contribute to neuronal excitotoxicity and neurodegenerative processes in AD [18,97,98,99,100,101,102,103,104]. Recently, Szepesi et al. also highlighted a crucial role of changes in microglia–neuron dialogue in AD [105], but Bukke et al. underlined the dual role of glutamatergic transmission in AD [104]. Recent data showed that activated microglia release glutamate together with ATP, which exerts an effect through stimulation of multiple receptor-mediated signaling, including purinergic receptors, such as P2X4 and P2X7 [106,107]. Moreover, ATP could be released from microglia by lipopolysaccharide (LPS), lysophosphatidic acid, and high intracellular calcium [Ca^2+^] level, and all these events may alter neurotransmission processes. Activation of glutamate receptors (GluRs) in microglia plays a significant role in the regulation of the inflammatory response and could be a promising target in therapeutic strategy in AD. In this neurodegenerative disease, the microglial release of glutamate may be evoked by secreted soluble Aβ precursor protein (sAPP) and Aβ peptides by TNF-α and may occur concomitantly with excessive stimulation of glutamate release from synaptic vesicles in the presynaptic part of nerve endings. The massive release of glutamate leads to over-stimulation of pre- and postsynaptic glutamatergic receptors, both ionotropic (AMPA, Kainate, NMDA) and metabotropic mGluR1 and mGluR5 (group I), mGluR2 and mGluR3 (group II), and mGluR4,6,8 (group III) [18,78]. Extrasynaptic glutamate receptors were suggested to significantly contribute to degenerative processes by triggering the expression of Tau protein and by suppression of transcription of the cAMP-response element binding (CREB) protein [103]. 

Dysfunction of clearance of glutamate together with its excessive release by microglia leads to excitotoxicity and aberrant extrasynaptic signaling and synaptic dysfunction, and then to synaptosis, neuronal death, and behavioral alterations [43]. Glutamate exerts pro- and anti-inflammatory effects depending on the type of receptor(s) that are involved, the amount of glutamate, and the type of cells engaged in the regulation of its homeostasis. Aβ peptides could be important triggers of glutamate release in AD. Additionally, the other toxic proteins such as α-synuclein (α-Syn), which accumulates in the neurons in 50–60% of AD cases, could be released into extracellular space by Aβ and may induce oxidative stress and additionally may exert an effect on glutamatergic signaling and cells death pathway(s) [53,54,108]. It has been known for a long time that GluRs in synapses play a crucial role in brain function in learning and memory and in the regulation of mood. However, the role of glutamatergic receptors (GluRs) in microglia in physiological conditions is not fully understood. It seems that GluRs in microglia are mainly responsible for the modulation of these cells′ response by altering their morphology and motility, generation of reactive oxygen species (ROS), and release of cytokines, leading probably to modification of neuronal function and synaptic transmission [109]. Microglia may influence glutamatergic neurotransmission mainly through AMPA receptor and presynaptic mGlu2/mGlu3 [110]. Recent studies have indicated the role of neuron–microglia interaction in the function and maturation of glutamatergic synapse and demonstrated how microglia influence synaptic organizations and activity [111,112]. Moreover, Perez-Rodriguez described the role of microglia in neurogenesis in the adult brain [113]. Despite all studies, the physiological function of microglial cells and their interplay with neurons in the brain is not fully elucidated. However, in neuroinflammation and in AD excessive glutamate and ATP release activate the extrasynaptic receptor (NMDA), purinergic P2 × 7 cation channel receptor and also other purinergic receptors, which could be consequently responsible for the atrophy of dendritic spines and for synaptic and neuronal degeneration [43,114]. Moreover, activated microglia may change tryptophan metabolism toward the kynurenine (KYN) pathway, which leads to an increase in the level of KYN, an antagonist of the NMDA receptor, and other molecules affecting glutamatergic signaling, such as quinolinic acid (QUIN), an agonist of the NMDA receptor (Figure 2) [115,116,117]. Furthermore, astrocytes produce more KYN, while microglia produce more QUIN [117]. The QUIN may evoke excitotoxicity even in physiological concentration [118,119]. The neurotoxic effect of QUIN may be additive or synergistic with neuroinflammation or excitotoxicity and can also evoke gliotoxicity [119,120,121].

It is postulated that an enhancement of glutamate level in the extrasynaptic area stimulates extrasynaptic NMDA receptor subunit GluN 2B, leading to alteration of Ca^2+^ ions homeostasis and to long-term depression (LTD), and consequently to dysregulation of synaptic function [104,122]. Moreover, these events may evoke alterations of APP expression and activation of the amyloidogenic pathway of APP metabolism in direction to Aβ liberation and oligomerization [104,123]. It is now accepted that Aβ oligomers are responsible for the alteration of synapses and for cognition impairment [124,125,126,127]. The synaptic function is highly dependent on microglia and astrocytes. Microglia cells constantly scan the brain milieu, neuronal cells, and synapses. Activated microglia are engaged in removing damaged neurons and altered–dysfunctional synapses, and this process is termed “synaptic stripping.” There are many factors by which microglia sense neurons and synaptic alterations/injury. Among them, chemokines and ATP play an important role. Moreover, the complement pathway, the part of the innate immune system, is significantly involved in microglia in the removal of pathogens and is also engaged in the elimination of synapses during neuroinflammation/neurodegeneration [128,129,130]. However, microglia concomitantly play a crucial physiological role in learning and memory by stimulation of novel learning-related synapse formation by the brain-derived neurotrophic factor (BDNF) receptor-mediated signaling pathway and probably also by other factors such as nerve growth factor (NGF) and glial cell-derived neurotrophic factor (GDNF) [27,129].

Microglial cells are significantly altered in brain aging and may contribute to age-associated cognitive alteration. Microglia senescence may be responsible for changes in synaptic communication and plasticity and consequently for alteration of cognitive functions. Microglia, through a variety of multiple receptors, including glutaminergic and GABA receptors and other receptors for neurotransmitters, neuropeptides, and neuromodulators, can sense neuronal activity. Microglia express adrenergic, dopaminergic, bradykinin, endothelin-1, histamine, and substance P receptors. Stimulation of these receptors evokes liberation of several cytokines and other bioactive compounds from microglia and affects the movement of these cells and phagocytosis [131]. In pathological conditions, microglia remove synapses from neuronal cells. However, the involvement of microglia in the brain depends on the type of pathology. For example, in prion disease, activation of microglia does not lead to synaptic stripping, but in AD this process probably could play an important role in some brain regions [132]. Additionally, the role of neurotransmitters in the modulation of microglial activity in AD is not elucidated. 

The previous data demonstrated that neurotransmitter level (GABA and glutamate) and Aβ, and also hyperphosphorylated Tau protein, are not significantly altered in the brain cortex, hippocampus, and retina at an early stage in the experimental model of AD (3×Tg mouse model) [133]. However, this study simultaneously demonstrated a few differential changes of microglia in retina and brain regions, which means different profiles in microglia branching at this early stage of AD (4-month-old AD-Tg mouse) with hypertrophy of microglia in the hippocampus and atrophic morphology of microglia in the retina. This and other studies did not answer the question, what kind of alterations of microglia could be evoked in AD pathology? Our recent data on the very early molecular changes in the AD-Tg mouse model indicated significant alteration in the expression of genes for enzymes involved in antioxidative defense and mitochondrial function [134]. The latest study by Styr and Slutsky [85] suggested that failures in firing stability and imbalance between firing homeostasis and synaptic plasticity in cortico-hippocampal circuits activate the driving force of early-phase AD, which may then lead to memory impairment.

## 6. GABAergic Signaling in Microglial Dialogue with Neurons in AD and Neuroinflammation

Alterations of glucose, glutamate level, and their metabolism may significantly affect gamma-aminobutyric acid (GABA), which is a major inhibitory neurotransmitter in the brain. Inhibitory neurons are crucial for the correct control and coordination of neuronal networks within and between various brain regions. The imbalance between excitation and inhibition, leading to variations in the activity of neural populations, was suggested to be a potential mechanism of cognitive dysfunction significantly contributing to the pathomechanism of AD [8,9,10]. 

The dysfunction of GABAergic signaling in human AD has been usually overlooked, even if impairment of inhibitory neurons and alterations in EEG, GABA levels, GABA receptors, etc., have been reported in AD patients and in experimental models (Table 2) [8,135]. 

However, the results did not give conclusive and consistent results. For example, studies on post-mortem levels of GABA, distribution, and activity of glutamic acid decarboxylase (GAD) often lead to contradictory results, likely because of significant limitations related to post-mortem analysis. Reduced GABA uptake due to loss of cortical and hippocampal GABA terminals in the AD brain was reported [142,143]. The levels of GABA receptors (GABA_A_, GABA_B_) and transporters (GAT1, GAT2, GAT3, BGT1) change in a region- and layer-specific manner [135,144]. In general, it seems that in AD an inhibition of GABAergic signaling in many brain structures occurs. 

However, the role of GABA in the brain appears much beyond the controlling function of neural circuits and networks, because glial cells, including microglia, are also GABAceptive cells. Microglia express GABA metabolism-related enzymes and functional ionotropic and metabotropic GABA receptors A–C [145,146,147,148]. However, some studies suggested that the effects of neurotransmitters (glutamate, GABA) on microglia may not be direct, because local application of neurotransmitters does not evoke electrical responses in microglia [149,150]. As an alternative, it was suggested that the effect is mediated indirectly via extracellular ATP and purinergic receptors [151].

Data on the effect of GABA on microglial activity are not consistent. It was demonstrated that GABA suppresses the reactive response of microglia to the proinflammatory stimuli, leading to inhibition of pathways mediated by nuclear factor kappa B (NF-κB) and p38 MAP kinase [146,147]. Moreover, studies on mouse cortex in vivo have demonstrated that ramified microglia respond to current levels of GABAergic neurotransmission, as a surface application of the GABA receptor blocker, bicuculline, significantly increases microglial processes motility [152]. On the contrary, GABA evokes activation of microglia via the nucleotide oligomerization domain (NOD)-like receptor family pyrin domain-containing 3 (NLRP3) inflammasome and NF-κB signaling pathways, leading to a significant increase in the mRNA and protein levels of TNF-α, IL-6, and IL-1β in microglial cell line BV2 [153]. 

The connection between neuronal activity and microglial activity in the pathomechanism of AD was demonstrated in 5×FAD mice, a well-established model of AD [154]. 5×FAD mice have reduced power in gamma oscillations during hippocampal sharp-wave ripples. Artificial stimulation of gamma oscillations in the hippocampus upregulated microglial genes related to phagocytosis, migration, and cell adhesion, evoked morphological transformation of microglia, increased co-localization of microglia and Aβ, and triggered Aβ peptide uptake by microglia. This effect was strictly dependent on GABAergic neurotransmission, because pre-treatment of 5×FAD mice with GABA_A_ antagonist, picrotoxin, completely canceled the beneficial effects of gamma oscillations. Therefore, GABA signaling seems to be essential for the activation of Aβ uptake by microglia.

## 7. Therapeutic Compounds Affecting Glutamate/GABA Neurotransmission in Microglial–Neuron Communication

In AD, the amyloid cascade hypothesis has gained a central position and dominated the field for the last three decades [5]. The fundamental principle of this hypothesis is that the accumulation of Aβ is the initial pathological event that triggers Tau pathology, mitochondrial dysfunction, neuroinflammation, and oxidative stress, leading finally to neurodegeneration and dementia [155,156,157]. However, AD is a multifaceted disorder with very complex pathomechanism, and its etiology remains not fully understood. Despite thousands of preclinical studies and clinical trials, the therapeutic possibilities are still very limited. No new treatment has been introduced into clinical practice since 2003. Therapeutic strategies based on an anti-Aβ approach have not yielded satisfactory results. The recent accelerated approval of aducanumab (a monoclonal antibody directed against soluble and insoluble forms of amyloid β) by the United States Food and Drug Administration (FDA) opened the floodgates to new disease-modifying drugs, but using aducanumab faced criticism and is considered controversial [158,159,160,161]. 

Early studies demonstrated that long-term use of nonsteroidal anti-inflammatory drugs (NSAIDs) declined the risk of developing AD [162,163,164]. However, the clinical trials on NSAIDs gave inconclusive results, showing protective effects, no effects, or even detrimental effects [165,166,167,168,169,170]. Data seem to suggest that anti-inflammatory therapy may be beneficial if introduced at an early stage of the disease, before symptom onset; however, it may be detrimental at a later stage, after cognitive impairment develops [169,170]. Additional factors, such as the rate of cognitive decline, type of NSAID (cyclooxygenase-2 inhibitor vs. non-specific), genetic background, general health, and lifestyle of the patient, may also be important.

Currently, acetylcholinesterase inhibitors and NMDA receptor antagonist are commonly prescribed medications for AD. Inhibitors of acetylcholinesterase (rivastigmine, donepezil, galantamine) have been approved for the treatment of mild and moderately severe AD. These drugs slow the development of the clinical symptoms, but cannot stop the progression of the disease. Memantine, an NMDA receptor antagonist, has been approved for the treatment of patients with moderate and severe disease. Moreover, combination therapy with acetylcholinesterase inhibitor and memantine has been proposed, but the clinical relevance of its effect is uncertain [171]. Memantine is used in the treatment of AD to inhibit NMDA receptor-mediated excitotoxicity. It preferentially acts on extrasynaptic NMDA receptors containing the GluN2A subunit [103]. Moreover, memantine inhibits the LPS-induced production of ROS and proinflammatory factors. Importantly, glia-mediated neurotrophic and anti-inflammatory effects of memantine are NMDA receptor-independent. Rosi and co-workers demonstrated in a rat model that memantine at therapeutically relevant doses inhibits the activation of microglia and reduces LPS-induced neuroinflammation [172]. In the model of Aβ toxicity in vivo, memantine significantly reduced changes of glial marker proteins and activation of inducible nitric oxide synthase (iNOS) [173]. The effect of memantine on inwardly rectifying K^+^ Kir2.1 channels was demonstrated in BV2 microglial cells, which was suggested to be one of the important mechanisms underlying memantine’s actions on microglial cells [174]. By contrast, memantine did not influence the activation of microglial cells in culture, suggesting that microglia–neuron communication is necessary for the anti-inflammatory effect of memantine [175]. Similarly, in rodent microglial cells stimulated with TNF-α, memantine did not affect the production of nitric oxide (NO), Ca^2+^ elevation, expression of inflammation-related genes, or phagocytic activity [176]. Memantine reduced Aβ-evoked phosphorylation of Tau protein in vitro [177]. In addition, memantine caused neurotrophic effects through the increased production of glial cell line-derived neurotrophic factor (GDNF) [178]. This effect on the GDNF level was evoked by inhibiting the activity of cellular histone deacetylase, leading to histone hyperacetylation. 

Other drugs that are NMDA receptor blockers, such as ifenprodil, fluoxetine, and desipramine, have not been approved for AD treatment. However, novel drugs are still developed to attenuate glutamatergic dysfunction in AD, among them riluzole. The clinical trial on riluzole (NCT01703117), an inhibitor of both glutamate release and postsynaptic glutamate receptor signaling, met the primary and the secondary outcome measures, showing a strong correlation between riluzole treatment, cognitive measures, and brain metabolism [179]. In transgenic mouse models of AD, riluzole modulated the activity of small conductance and Ca^2+^-activated K^+^ channels (SK channels), decreased Aβ levels (oligomers and plaques) and Tau pathology (total level and phosphorylation), and reversed changes in gene expression [180,181,182]. Importantly, analysis of gene expression patterns showed that riluzole impacts some immune-related pathways implicated in AD, including expression of canonical gene markers for microglia (e.g., Clec7a and IRF7) [181]. Another compound, BMS-984923, an mGluR5 silent allosteric modulator, is now in a phase I trial (NCT04805983). The action of BMS-984923 is not limited to modulating mGluR5-dependent calcium signaling. It recovered Aβ oligomer-dependent impairment of intracellular signaling, synaptic plasticity, Tau pathology (total level and phosphorylation), and synaptic loss in a mouse transgenic model of AD [183]. However, this drug did not impact Aβ load and microgliosis. Therefore, these glia-related effects of novel pharmacological compounds targeting glutamate signaling should be considered and studied.

Additionally, the clinical application of GABAergic drugs should be further evaluated for the treatment of behavioral and psychological symptoms of dementia in AD, as it was mentioned many years ago [184]. The positive GABA_A_ receptor modulating steroid allopregnanolone (APα) is currently in a phase II trial (NCT04838301). In a single dose, APα was shown to reduce Aβ generation and to promote regeneration of neurons and restore learning and memory in a 3×Tg mouse model of AD [185,186], but the chronic treatment caused memory decline in wild-type mice and accelerated dementia in selected AD mice models [187,188]. Interestingly, APα affects microglial morphology and phagocytic function, which might potentially contribute to its beneficial effect in AD [189]. It cannot be excluded that mechanisms other than modulating glutamatergic and GABAergic signaling contribute to the neuroprotective effects of the compounds described above. Moreover, current strategies and novel drug approaches for AD were described, including modulatory effects of autophagy on APP processing, as a potential treatment target for AD [190,191,192] (Table 3).

As mentioned in this review, the metabolism of glutamate and GABA is very complex. Glutamate links carbohydrate and amino acid metabolism via the tricarboxylic acid (TCA) cycle and plays a key role in maintaining nitrogen and ammonia homeostasis in the brain [193,194]. Moreover, glutamate through its receptor-dependent signaling is also involved in APP/Aβ and protein Tau metabolism. However, several recent studies have indicated that alterations of APP/Aβ and Tau cannot fully explain the pathomechanism of the sporadic form of AD [195]. Using quantitative metabolomics, proteomics, and lipidomics methods to analyze plasma and cerebrospinal fluid (CSF) indicated specific and close association of amino acids (including homocysteine, a non-proteinogenic amino acid) and tryptophan metabolites (kynurenic acid and quinolinic acid) with AD biomarkers: Aβ42, Tau, and phospho-Tau (Thr181). Importantly, the peripheral metabolites of the kynurenine pathway were recently suggested as potential biomarkers in neurodegenerative diseases, including AD [196]. These advanced methods could be helpful in better understanding the AD pathomechanism and in the identification of novel promising targets in therapy [197,198]. It has been proposed that metabolic impairment, including alterations of glucose metabolism, and modifications of amino acids and proteins can significantly enhance the risk of AD. Moreover, a growing body of data demonstrated the failure of mitochondrial dynamics and function in AD and alterations of glucose metabolism in the TCA cycle and in the glycolytic pathway, shifting it from oxidative phosphorylation to aerobic glycolysis [199,200]. Dyslipidemia, hypertension, abdominal obesity, and insulin resistance, which alter glucose metabolism, are accepted hallmarks of metabolic syndrome (MetS) and potential causes of AD [201]. Accordingly, type 2 diabetes mellitus-related insulin and obesity-related adiponectin have been proposed to be promising targets in the therapy of AD [192,202,203]. Several compounds improving mitochondrial function or glucose metabolism are currently in AD drug development, including metformin (NCT04098666) and tricaprilin (NCT04187547) in phase 3, and benfotiamine (NCT02292238), dapagliflozin (NCT03801642), liraglutide (NCT01843075), and S-Equol (NCT03101085) in phase 2. Some of these compounds, such as metformin, which is an insulin sensitizer and improves CNS glucose metabolism, may potentially affect glutamatergic and GABAergic signaling in AD, including microglia–neuronal cross-talk [204,205].

The compounds affecting systemic and brain metabolic disturbances together with modulators of glutamatergic, GABAergic, and other neurotransmitters-related signaling pathways could improve the therapeutic efficacy against AD. 

## 8. Concluding Remarks and Future Challenges

The recent available data support the crucial role of microglia–neuron communication in the brain and highlight the view regarding the microglia cells as the guardians of the central nervous system in physiological conditions and as important players in AD pathology. This review summarizes the data on alterations of glutamate and GABA level and signaling in microglia–neuron interaction at the early stage of AD and in the progression of this most severe dementia. However, the limitation of this article is the insufficiency of published data, which is related to the low availability of high-quality post-mortem human samples and limited relevance of current animal models of AD [206]. Moreover, some studies present contradictory results. Analysis of samples with low post-mortem interval (PMI) from various brain structures, and various disease stages, should reduce discrepancies and enable a better understanding of the complex interplay between cells and between neurotransmitter systems in AD [135]. An additional source of bias in data concerning glutamatergic and GABAergic systems in AD is the fact that drugs that are commonly prescribed in AD impact neurotransmission, potentially including glutamatergic and GABAergic systems. Therefore, many basic questions remain not elucidated. 

The fundamental question arises if the effect of glutamate and GABA on microglia is direct or is mediated by ATP and its action on several purinergic receptors. The other question that needs an answer is related to the role of ATP, concomitantly secreted with these both neurotransmitters. The question is, how may ATP modulate/alter the communication between neurons and microglia in AD? The following important question is if several microglial receptors for neurotransmitters, neuropeptides, and neuromodulators are activated during synaptic transmission and how they change in AD. Then the question arises according to the involvement of the kynurenine pathway in modulation of glutamatergic signaling and microglia–neuron communication in AD. Moreover, the role of alterations of microglia–neuron cross-talk in disturbances of cognition function should be better elucidated. 

To answer these questions, a more rigorous and systematic analysis of data from human samples is necessary. Advanced non-invasive spectroscopic and imaging techniques with future improvements will provide the most adequate data on the impairment of metabolic processes and neurotransmission in AD [207,208]. In correlation with novel serum biomarkers, they will be useful for the complex evaluation of individuals at various stages of AD. There is also a need for more relevant animal models that could provide a mechanistic explanation for the impairment of glutamatergic and GABAergic signaling. Moreover, because of some differences in human and rodent physiology, advanced models in vitro could offer additional benefits.

Answering and elucidating these several questions is necessary for better understanding the dialogue between microglia and neurons in neuroinflammation and in the pathomechanism of AD and will be fundamental for elaborating better strategies in the diagnosis and therapy of AD. 

## Figures and Tables

**Table 1:** Alterations of glutamatergic system components in human AD.

Glutamate Components	Localization	Expression	References
Transporters			
EAAT2	Presynaptic	↓	[93]
EAAT3	Postsynaptic	↓	[94]
Ionotropic receptors			
NMDA	Post- and extrasynaptic	↑ ↓	[94,95]
AMPA	Pre-, post-, and extrasynaptic	↑ ↓	[94,96]
KA	Pre-, post-, and extrasynaptic	↓	[94]
Metabotropic receptors			
mGluR3 (group II)	Pre- and postsynaptic	↑	[96]

EAAT2/3—excitatory amino acid transporter 2/3, NMDA-N-methyl-D-aspartate, AMPA- alpha-amino-3-hydroxy-5-methyl-4-isoxazolepropionate, KA-kainate, ↓/↑-decreased/increased expression.

**Table 2:** Alterations of GABAergic system components in human AD.

GABA Components	Localization	Expression	References
Transporters			
GAT1	Pre-, post-, and extrasynaptic	↓	[136]
GAT3	Pre- and extrasynaptic	↓	[135,136]
BGT1	Extrasynaptic	↑	[136]
Ionotropic receptors			
GABA_A_	Pre-, post-, and extrasynaptic	↓	[137,138,139]
Metabotropic receptors			
GABA_B_	Pre-, post-, and extrasynaptic	↓ ↑	[140,141]

GAT1/3-GABA transporter 1/3, BGT1-betaine/GABA transporter 1, ↓/↑-decreased/increased expression.

**Table 3:** Pharmacological compounds affecting glutamatergic/GABAergic signaling in Alzheimer’s disease.

Compound	Effect on Glutamatergic/GABAergic Signaling	Non-Canonical Effects
Memantine	NMDA receptor antagonist	Inhibition of LPS-induced production of ROS and proinflammatory factors
		Inhibition of microglia activation and neuroinflammation
		Inhibition of Aβ-evoked iNOS activation and expression of glial marker proteins
		The effect on inwardly rectifying K^+^ Kir2.1 channels in microglial cells
		Inhibition of Aβ-evoked phosphorylation of Tau protein
		Inhibition of the activity of cellular histone deacetylase leading to histone hyperacetylation and consequently to increased production of GDNF
Riluzole	An inhibitor of both glutamate release and postsynaptic glutamate receptor signaling	Modulation of the activity of small conductance, Ca^2+^-activated K^+^ channels (SK channels)
		Decrease in Aβ levels (oligomers and plaques)
		Decrease in Tau pathology (total level and phosphorylation)
		Modulation of the gene expression patterns (including immune-related pathways implicated in AD)
BMS-984923	The allosteric modulator of mGluR5 receptor	Inhibition of Aβ oligomer-dependent impairment of intracellular signaling
		Decrease in Tau pathology (total level and phosphorylation)
Allopregnanolone (APα)	The positive GABA_A_ receptor modulating steroid	In a single dose: decrease in Aβ generation
		In a single dose: neuroregeneration
		In a single dose: improvement of learning and memory
		In a chronic treatment: memory decline and accelerated dementia
		Modulation of microglial morphology (protrusions extension)
		Decrease in migratory capacity of microglia
		Decrease in phagocyting activity of microglia

LPS-lipopolysaccharide, ROS-reactive oxygen species, Aβ-amyloid β, iNOS-inducible nitric oxide synthase, GDNF-glial cell line-derived neurotrophic factor.

## Data Availability

Not applicable.
